# Flexible Assertive Community Treatment in Rural and Remote Areas: A Qualitative Study of the Challenges and Adaptations of the Model

**DOI:** 10.3389/fpubh.2022.913159

**Published:** 2022-07-22

**Authors:** Kristin Trane, Kristian Aasbrenn, Martin Rønningen, Sigrun Odden, Annika Lexén, Anne Signe Landheim

**Affiliations:** ^1^The Norwegian National Advisory Unit on Concurrent Substance Abuse and Mental Health Disorders, Inland Hospital Trust, Hamar, Norway; ^2^Inland Norway University of Applied Sciences, Hamar, Norway; ^3^Health Sciences, Lund University, Lund, Sweden; ^4^The Norwegian National Advisory Unit on Concurrent Substance Abuse and Mental Health Disorders, Inland Hospital Trust, Inland Norway University of Applied Sciences, Hamar, Norway

**Keywords:** rural, remote, mental health, flexible assertive community treatment (FACT), innovation, modification

## Abstract

**Background:**

Flexible assertive community treatment (FACT) is an innovative model for providing long-term treatment to people with severe mental illness. The model was developed in the Netherlands but is now used in other countries, including Norway, which has a geography different from the Netherlands, with many rural and remote areas. Implementation of innovations is context dependent. The FACT model's potential in rural and remote areas has not been studied. Therefore, we aimed to gain knowledge regarding the challenges and modifications of the model in rural and remote contexts and discuss how they can affect the model's potential in such areas. This knowledge can improve the understanding of how FACT or similar services can be adapted to function most optimally in such conditions. We sought to address the following questions: Which elements of the FACT model do team leaders of the rural FACT teams find particularly challenging due to the context, and what modifications have the teams made to the model?

**Methods:**

Digital interviews were conducted with five team leaders from five rural FACT teams in different parts of Norway. They were selected using purposive sampling to include team leaders from some of the most rural teams in Norway. The interviews were analyzed using thematic text analysis.

**Results:**

The following three themes described elements of the FACT model that were experienced particularly challenging in the rural and remote context: multidisciplinary shared caseload approach, intensive outreach and crisis management. The following eight themes described the modifications that the teams had made to the model: intermunicipal collaboration, context-adaptive planning, delegation of tasks to municipal services, part-time employment, different geographical locations of staff, use of digital tools, fewer FACT board meetings, and reduced caseload.

**Conclusions:**

Rural and remote contexts challenge the FACT model's potential. However, modifications can be made, some of which can be considered innovative modifications that can increase the model's potential in such areas, while others might move the teams further away from the model.

## Introduction

It can be challenging to provide good health services in rural and remote areas (1–11), because of long travel distances (2, 12–15) and low population densities (12, 16). Flexible Assertive Community Treatment (FACT) is a multidisciplinary recovery- oriented model used to provide long-term outreach care and integrated treatment to people with severe mental illness (17). The FACT model was developed in and adapted to the Dutch context (17, 18). However, it is currently used in other countries (19–26), including in rural and remote parts of Norway (27), where the model is a completely new way of organizing care, and thus an innovation (28). Mental health services are affected by both geography and the service system (29). Norway has a complex and fragmented mental health service system (27, 30), and adaptions of the FACT model to the system has been made (30). Prior to the establishment of FACT teams, several different services provided treatment and care to the target group (31, 32), such as inpatient and outpatient specialist health services, Mental health and substance abuse services in primary care, The Norwegian Labor and Welfare Organization (NAV), and General practitioners (GP). Norway has a completely different geography from the Netherlands. Approximately one-fifth of the Norwegian municipalities have fewer than 2,000 inhabitants (33). The population density is 15 inhabitants per km^2^ (34), and half of the population lives in regions with more than 50,000 inhabitants (35). By contrast, the population density of the Netherlands is 508 inhabitants per km^2^ (34), and 80% of the population lives in regions with over 50, 000 inhabitants (35). Such differences make it challenging to replicate an innovation to a new context (36). The implementation of innovations is context-dependent (28, 37–39) and must be adapted to local conditions (28). Thus, a rural and remote area, henceforth referred to as rural, with very long travel distances and very low population density can affect the potential of the FACT model.

The FACT model is a further development of the Assertive Community Treatment (ACT) model, which was developed in the US to provide treatment to 20 % of patients with the most severe mental illness (40). The ACT model is proven to be less appropriate in rural areas (41–44). This is one of the main reasons for developing the FACT model in the Netherlands (45). The FACT model has a broader target group than ACT and includes all patients with severe mental illness. A key element of the FACT model is the multidisciplinary shared caseload approach. On the one hand, this means that the FACT teams must be multidisciplinary, including staff members with backgrounds in health care and social work, such as psychiatrists, nurses, social workers, psychologists, substance abuse specialists, employment specialists, and peer specialists. Moreover, these staff members should have almost full-time employment. On the other hand, the multidisciplinary shared caseload approach implies that the expertise of the various staff members must be used actively in patient care and the staff must not work as only individual therapists but as a team actively using each other's different competencies. As part of this approach, the teams have daily meetings, called FACT- board meetings, to discuss and co-ordinate care and support needs. In these meetings the teams also discuss how intensive care patients need, intensive ACT treatment or shared caseload approach (17). According to the FACT fidelity scale 2010 (46), patients receiving shared caseload approach are supposed to meet at least 4 staff members in a year. When patients get intensive ACT treatment, all team staff are supposed to know and work with the patients, and a goal is that each patient have met more than 3 staff members in 2 weeks. Other key elements of the model are intensive outreach, crisis management, and the provision of most services by the team. Generally, the teams have a base location where all staff members meet daily. The catchment area are recommended to be around 50,000 persons over the age of 18 years, and the caseload, which is the number of patients per staff member, are recommended to not exceed 20 (17). Even though the FACT model is developed to function in more rural regions than the ACT model, the Norwegian rural context might cause challenges and adaptions that differs from those in the Netherlands. Therefore, it can be difficult to comply with these requirements in a so rural areas as one can find in Norway.

The rural characteristics of long distances and low population density might create challenges. Long travel distances can create transport challenges (2, 8, 47), and make it more difficult to handle crises (48) and perform outreach work (13, 15, 49–51). This can be further complicated by challenging driving conditions (11, 15) because heavy snowfall is common in Norway. Long travel distances can lead to time wastage (14), and increased costs (1, 14). Sparsely populated areas can lead to fewer (1, 4, 47, 52) and less specialized services (47), poorer access to professionals (2, 4, 6, 16, 47, 52–57) and specialist expertise (4, 12) as well as high staff turnover (54, 56). Multidisciplinary teams can operate in rural areas (9, 58), but care and treatment models are often developed for urban contexts (9, 51, 52). In some cases, considerable modifications are made to such models in rural contexts (14, 43, 49, 51, 52, 59), as mentioned also to the ACT model. Therefore, FACT teams have been used instead of ACT teams in some places (60), including in Norway, where several FACT teams have been established in rural areas. However, knowledge about how these characteristics affect the potential of the FACT model in very rural contexts, is limited. Even though the FACT model is developed to be adapted to local contexts (45), the discrepancy between the FACT model and rural conditions can affect the model's potential. In such situations, modifications can be made by adding, reducing or removing elements in the model (36). If the modifications are large, they may weaken the model. However, if they fit in well with the model, and are developed into new practices that become established as a new way of working and can be repeated, they can be considered as innovations according to Toivonen (61). Adaptations in rural areas can be intermunicipal collaboration (62), part- time employment (63), fewer internal meetings (49, 51), lower caseloads (51), digital tools (13, 14, 52, 56, 64, 65), good planning (13, 15), flexible working methods (66), and collaboration with other services (63, 67, 68). Limited information is available regarding the modifications made to the FACT model by rural FACT teams and if such modifications work.

Therefore, this study aimed to gain knowledge about the challenges and adaptations of the FACT model in rural contexts and discuss how these can affect the potential of the model in such areas. This knowledge can improve the understanding of how the FACT model or similar models can be adapted to function most optimally in rural conditions. Therefore, we sought to address the following questions: Which elements of the FACT model do team leaders of rural FACT teams find particularly challenging due to the context, and what modifications have the teams made to the model?

## Methods

### Design

This study had a qualitative design (69, 70). To provide a rich and detailed picture of experiences (71), individual interviews with team leaders of rural FACT teams were conducted.

### Study Setting and Sample

Using purposeful sampling (71), five team leaders from some of the most rural FACT teams in Norway were recruited. These teams were located in different parts of Norway, but all of them worked in regions with long travel distances and low population densities. The teams were established between 2014 and 2020. All teams were organized in specialist health care and had a binding collaboration agreement with the municipalities involved. Most teams had shared employer responsibility between the primary and specialist health care services. The team's total caseload varied between 16 and 40 patients. The individual caseload varied between 4 and 12 patients per team staff. Norwegian FACT teams use the FACT Fidelity scale from 2010 (46). Fidelity measurements have been carried out in all five FACT teams, and they would all have been certified in the Netherlands. The number of staff, FACT team members, varied between 8 and 12. All teams had part- time positions, from one to everyone. Three teams had more than one physical location. More information on the characteristics of the FACT teams is presented in Table 1.

**Table 1:** Characteristics of the FACT teams included in the study.

	**FACT team 1**	**FACT team 2**	**FACT team 3**	**FACT team 4**	**FACT team 5**
Organization	Specialist health care	Specialist health care	Specialist health care	Specialist health care	Specialist health care
Shared employer responsibility between primary and specialist health care services	Yes	Yes	No	Yes	Yes
Binding collaboration agreement between primary and specialist health care services	Yes	Yes	Yes	Yes	Yes
Total caseload	29	33	40	18	16
Number of team members	10	10	8	8	12
Number of part- time employments	1	3	4	8	12
The team had more than one physical location	Yes	Yes	No	No	Yes
Total population of catchment area	23,500	18,500	19,500	17,500	5,000
Size of catchment area in square kilometers	1,200 km^2^	1,300 km^2^	4,300 km^2^	5,400 km^2^	3,000 km^2^

Team leaders were interviewed because they were regarded as having the best knowledge of the teams' challenges and modifications under rural conditions. They knew the FACT model well and their work involved both direct contact with patients and being responsible for the team's daily work. In four of the teams, the team leaders were employed in specialist health care, while the leader of the fifth team was employed in primary care.

### Data Collection

In-depth individual interviews were conducted in September 2021. Because of the coronavirus disease 2019 (COVID-19) pandemic, the interviews were conducted online. The interviews had a semi-structured approach (70, 71). The interview guide focused on participants' experiences of how the FACT model functioned in rural areas, with the following three main topics: descriptions of the specific challenges, the way they handled the challenges and their experiences of their modifications of the FACT model. The first author conducted the interviews, which lasted between 55 and 60 min.

### Data Analysis

The interviews were transcribed verbatim by the first author, and analyzed according to Braun and Clarke‘s thematic text analysis (71, 72). The analysis was largely data-driven, but the data were sorted in accordance with the central elements of the FACT model. First, the interview recordings were heard, and the transcripts were carefully read to identify patterns. Initial thoughts were written down. Then, the whole data set was initially coded line by line; all challenges, modifications and experiences of modifications were coded. Codes were named according to their content. Thereafter, themes were generated. This process was guided by the described challenges and modifications related to the central elements of the FACT model, omitting challenges and modifications that were not specific to rural areas. The transcripts were then re-read to ensure that the themes were supported by the data. This led to several changes in the names of codes and themes. To ensure credibility, data were scrutinized for exceptions to codes and themes (69), and some exceptions were found. The initial thoughts were then read to check how they matched the themes and codes, which they largely did. The focus in the initial thoughts was on whether the teams appeared to be working in accordance with the FACT model. In the process of writing, previous stages were returned to, and transcripts were re-read to ensure all relevant data were included and that the selected quotations were the ones describing the content best.

### Ethical Approval

The study was approved by the Data Protection Officer for South-Eastern Norway (ID 15459224). The consent letter contained information about the study aims and data storage and how confidentiality and anonymity were ensured.

## Results

The following three themes were identified to describe the elements of the FACT model that were experienced as particularly challenging due to the rural context: multidisciplinary shared caseload approach, intensive outreach and crisis management. The following eight themes were identified to describe modifications made by the teams: intermunicipal collaboration, context-adaptive planning, delegation of tasks to municipal services, part-time employment, different geographical locations of staff, use of digital tools, fewer FACT board meetings, and reduced caseload.

### Elements of the FACT Model Found to Be Particularly Challenging in the Rural Context

#### Multidisciplinary Shared Caseload Approach

Most team leaders described big or small challenges in the access to multidisciplinary expertise, in the form of e.g., psychiatrists, psychologists, employment specialists or peer specialists. Some team leaders also mentioned a rather high turnover in the team. One said that this particularly applied to psychiatrists and explained: “For a very long time it's been like this. We've had a psychiatrist for 2 weeks, and then a new one comes.” Most team leaders described how long travel distances took up so much time that they were sometimes unable to use the multidisciplinary shared caseload approach to the extent recommended by the FACT model. Some leaders said that decisions about who would meet which patients were sometimes based more on geographical practicalities than actual needs. Many leaders said that this meant that they sometimes worked more as individual therapists than that recommended by the FACT model, thereby reducing the shared caseload. One explained: “We probably work more as individual therapists than we should according to the model. So, unfortunately, we can't completely follow the model in that respect”. Some leaders also found it challenging to hold the daily multidisciplinary FACT board meetings, especially because the long travel distances took up so much of their time.

#### Intensive Outreach

Most team leaders described how long travel distances made outreach care challenging, especially when there was a need for intensive follow-up care. Time taken to travel to the patients who lived farthest from the team base varied between the teams, from 45 min to 2.5 h one way. One leader said: “…and of course, the thing is that we've got no chance to provide intensive treatment to all patients in an unstable phase. We can't travel three or four times a week to patients who are 2.5 h away”. They also found it difficult to ensure that the FACT team provided most of the services. Establishing contact with patients was also a challenge, as one leader said:

*Then you have the ones that are hard to establish contact with. It*'*s easy to get in your car and drive 10 min to knock on a patient*'*s door, but what about driving for an hour to knock on a door you*'*re pretty sure won*'*t be opened*.

#### Crisis Management

The team leaders said that there were few other health services in the region and the inpatient facilities were far away. Many said that this made crisis management more challenging, especially in cases of involuntary admission. The challenges involved not only the team's ability to rapidly deal with crises themselves but also access to the police and ground and air ambulances. These were described as services that took too long to arrive, which meant that the teams often had to wait with the patient for a long time, and one team leader said: “its unworthy for the patients.” One team leader described waiting for the police: “…and then we just had to sit here, up to 6 or 7 h and wait for them to come and pick up the patient.”

### Descriptions of Adaptations of the FACT Model

#### Intermunicipal Collaboration

To have an adequate number of patients, agreements on intermunicipal collaboration were signed when the FACT teams were established. The number of municipalities involved varied between the teams, from two to six. This was described as a necessary modification. However, team leaders of the FACT teams where more than two local authorities were involved found this time-consuming because the teams needed to become familiar with and handle differences between the local authorities with regard to aspects such as organization, structures, procedures, digital systems, tools and approaches. One leader said: “We have to work with several different municipalities, and they have slightly different approaches and do things in slightly different ways. That's a challenge, and we spend a lot of time providing information”. This was described as time-consuming and requiring good communication skills. Another leader said:

*We have to provide information to many different service providers in the various municipalities. We have to be very adaptable and get to know what services they have and how they are structured. It*'*s quite an effort, and you have to be really keen on co-operating to make it work*.

#### Context-Adaptive Planning

The team leaders described how the context required adaptive planning, especially to address the distances, multidisciplinary shared caseload approach and crises. Plans had to be well thought out and flexible. Some leaders reported having made crisis plans to have a clear idea of what they and other service providers should do in crises. The teams had to spend considerable time driving, sometimes on bad roads, with snow, stormy weather, closed bridges or ferries. Many leaders said that they had too few cars and good planning was needed to distribute the cars among the team staff. One explained: “So, I think having enough cars at all times is essential to make this work properly.” Many leaders also said that they visited several patients in one journey when they had to drive a long way. One of them said: “There are days when we go out to four or five patients, and especially when it's a long drive, we have a schedule for who to visit.” Most team leaders also mentioned that the team staff used the time in the car for co-ordination, internal meetings and planned and unplanned conversations. One explained: 'We have very little downtime in the car. It's mostly used for telephone consultations, collaboration meetings or internal meetings. For example, we often have FACT board meetings in the car.” Some leaders said that they focused on using resources effectively because all the planning consumed a lot of time.

#### Delegation of Tasks to Municipal Services

The team leaders described how the teams, to a greater or lesser extent, handled the long travel distances by collaborating with municipal services to delegate different tasks. One explained: “It would be very difficult for our team to provide all the services, precisely because of these long (travel) distances.” All leaders described how they needed help from home care services to deliver medicines, and they had delegated some or all of the medication deliveries to them. In cases where the distances were particularly long, the teams also collaborated with primary home care or mental health and substance abuse services for daily follow-up care, crisis management or driving patients for admission to the hospital. One leader said that they delegated a lot of work to the municipalities during patients' good periods, while another felt that they needed primary care particularly in patients' more difficult periods. One leader explained: “We really need home care services to provide part of the care in the good periods.” Some leaders also mentioned that the FACT team had the overall responsibility and co-ordinated the delegation of tasks to the municipal services.

#### Part-Time Employment

Most leaders said that one or more team staff members worked part-time in the FACT team. This was because of challenges in getting access to the needed expertise. However, some found this to be challenging, time-consuming and contrary to the multidisciplinary shared caseload approach. One of them explained:

*We see it in our meetings, you have to repeat the same thing for several days because not everyone gets the information from day to day. So, you spend extra time on meetings to make sure everyone is up to date. So, we see that part-time positions are bad for continuity*.

When asked about what he thought of part-time work, one team leader replied: “I don't like it.” He felt that 80% of a full-time job would be fine and that it should be the minimum. Lower percentages led to poorer communication between the team staff. In one of the teams, everyone worked part-time, which the leader described as “challenging” and “exhausting.” He said that they were often torn between their work in the FACT team and other parts of the service system within the mental health services: “We just offer a kind of piecemeal service.”

#### Different Geographical Locations of Staff

Some leaders reported coping with the problem of long travel distances by having more than one base; thus, the staff members were in the municipality to which they belonged. This was described as enabling more intensive outreach work, involving less task delegation, making it easier to handle crises and saving time. One leader explained: “If the whole team had met more often, we might have spent several days' work just driving.” One team was working on hiring someone to be located elsewhere, and the leader said: “If something crops up, he could deal with it much faster. So, we're trying to get staff who are closer to hand if there's a crisis somewhere.” The team leaders whose staff members worked physically at the same place described plans for combining homeworking with working in the office to a greater extent and said: “If you live close to where you're going to have the first meeting with a patient, there's no point driving 1 hour to get to work and then 1 hour back again”. Some leaders thought that not meeting face-to-face every day worked well, while others said that it could negatively affect the team feeling because it left fewer opportunities for short questions and debriefings. Some leaders said that they focused on this and consciously worked toward preventing divergent practices and loss of team spirit by providing advice, guidance and regular face-to-face meetings. One said: “We've been very much aware about working in a multidisciplinary manner. We don't want two different teams in one team.”

#### Use of Digital Tools

All team leaders reported using digital tools to some extent, both internally and externally. Some reported having virtual FACT board meetings, and web conferencing was sometimes used for treatment and transfer meetings. Some leaders also said that online sessions with patients could be held in some cases as a supplement to face-to-face meetings. The frequency of such contacts depended on the patients' wishes, state of health and access to the internet. Digital tools were also described as facilitating the use of the diverse expertise in the team, such as when a patient had a consultation with several team members. This would take the form of a hybrid meeting, where one team staff member would be physically present with the patient and connect digitally with the other staff members. One leader explained: “We can't be four staff traveling long distances, so maybe then one of us would go, and the others would be connected digitally.” The team leaders also stated that the COVID-19 pandemic had provided better opportunities for the use of digital tools, because of a lower threshold for use and improved technical solutions in the services. One leader explained: “I think COVID-19 made the municipality take action to improve our systems. So, it's got a lot better, and now we have equipment that works when we need it.”

#### Fewer FACT Board Meetings

As mentioned earlier, some leaders found it challenging to hold the daily multidisciplinary FACT board meetings, especially because the long travel distances took up so much of their time. Two team leaders stated that they, therefore, had reduced the frequency of the FACT board meetings to twice a week. One said: “We can't spend too much time in meetings.” This was described as deviating from the FACT model, as one leader explained: “We've had to make some structural changes, obviously at the expense of team feeling, methodology and updates from the whole team. We go straight out to our patients and have whole team meetings twice a week.”

#### Reduced Caseload

The team leaders felt that the time pressure as described above made it impossible to cope with a caseload of almost 20 patients per team staff member. One explained: “It's a challenge to have the portfolio of patients described in the model. I don't think that's possible for a rural team. Then you could only skim the surface.” All team leaders stated that they had therefore decided to decrease the caseload, which varied from four to 12 patients per team staff member. One of them said that lower caseloads made it easier to follow the FACT model and explained: “If you want this model, well then you may need a smaller number of patients to be faithful to the model.” Some reported feeling pressurized by the management to increase their caseloads.

## Discussion

This study indicates that rural contexts such as those you can find in Norway, challenge the potential of the FACT model. The FACT teams appeared to be in a dilemma wherein they attempted to follow the model but had to make local modifications. Some of these modifications appeared to increase the potential of the FACT model in rural areas and could be considered innovative modifications. Other adaptations meant that the teams' work was less in line with the FACT model. We first discuss how the rural context challenges the model's potential and then the innovative modifications.

### The Rural Context Challenges the Potential of the FACT Model

The FACT teams experienced many of the same challenges as other rural services. One of the key components of the FACT model is the multidisciplinary shared caseload approach. This was difficult to achieve because of low population densities and long travel distances in rural areas. Most team leaders described problems in accessing expertise and dealt with part-time employment. Although this is probably an inevitable modification in many rural teams, the FACT model does not recommend many part-time positions because this can compromise continuity and the shared caseload approach, and too much time can be spent on information exchange (17). The team leaders also described this issue. In addition, if the teams have challenges with high turnover, do not have daily FACT board meetings and do not use the different professional groups in their outreach work, as some team leaders described, there is a risk that the shared caseload approach will be further hampered. The FACT model places great emphasis on this approach and highlights that the different disciplines must work together on a daily basis, have professional discussions and use the different forms of expertise actively in patient care and treatment (17). Thus, part-time positions and fewer FACT board meetings appear to be modifications where an element in the model is reduced, resulting in making the multidisciplinary shared caseload approach more difficult to accommodate.

Intensive outreach, crisis management and self-provision of most services are other core elements of the FACT model. The long travel distances to patients and facilities made the accomplishment of these elements particularly difficult. Studies of ACT teams (49, 50), and a Danish study of FACT teams (13) also found that travel distance can affect the intensity of outreach care. Difficult driving conditions can further complicate this issue. Delegation of tasks to primary care appeared to be a modification that maintained outreach intensity to some extent and improved crisis management. However, this means that the FACT teams must collaborate with other services and co-ordinate care they do not provide themselves, which might be challenging (30). Therefore, a high degree of delegation of tasks to other services can make the teams depart further from the FACT model, which specifies that other services should be used as little as possible to ensure a good overview and co-ordination (17). Neither urban nor rural Norwegian FACT teams provide all services themselves and co-ordination and collaboration are needed (30). This appears to be reinforced by the rural context, where even more services are provided by others. Community mental health services need to collaborate with multiple service providers (29). This emphasizes the importance of collaboration in both urban and rural FACT teams, implying the need to increase the focus on service system collaboration in the FACT model. However, a study of patients of urban and rural FACT teams reported that the patients experienced FACT to be better than their earlier treatment because the FACT teams to a large extent provided the services they needed (73). Nevertheless, one challenge that remains is the remoteness in relation to crisis management. The long waiting times for emergency services cannot be solved by the teams themselves. Thus, it appears to be difficult for the rural FACT teams to meet the requirements in the FACT model regarding intensive outreach work, crisis management and providing most services themselves. Concurrently, some delegation of tasks to the municipal services is a modification that might work, as long as the teams have a good knowledge of the services and the ability to co-ordinate and collaborate with them. However, there are fewer services in rural regions, and thus communication between professionals might be easier (4), which might facilitate co-ordination when delegating tasks.

### Innovative Modifications Can Increase the Potential of the FACT Model in Rural Areas

Intermunicipal collaboration and lower caseload appear to be prerequisites for FACT teams to function in rural areas such as those in Norway. To achieve a large enough population base, local authority collaboration is a crucial modification wherein a new element is added to the FACT model. Nevertheless, the teams have far fewer inhabitants in their catchment areas than that recommended by the FACT model, and intermunicipal collaboration increases the travel distances. Thus, the population density will still be low, which means that the rural FACT teams in Norway face challenges similar to those described in the rural areas in other studies (1, 2, 4, 6, 12, 16, 47, 52–57). Many factors increase the time pressure, including intermunicipal collaboration, additional planning, part-time positions and long travel distances. All these factors affect the number of patients that can receive care and treatment, and the teams, therefore, have a reduced caseload. This has no direct impact on patient care but could make the teams less cost-effective, which in turn could affect whether the rural FACT teams are considered sustainable over time. Nevertheless, intermunicipal collaboration and lower caseloads appear to be innovative modifications that may increase the potential of the FACT model in rural areas because they can be established in practice and repeated by other FACT or similar teams.

Challenges involved in intensive outreach and the multidisciplinary shared caseload approach can apparently be reduced to some extent using digital tools. This can be a useful modification wherein one adds an element to the model. Studies have shown that ACT (14) and FACT (13) teams can mitigate the problem of long travel distances using digital meetings, including in situations of crisis management (14). Both the present study and another Norwegian study (74), described how virtual tools increased in the FACT teams because to the COVID-19 pandemic, and one might state that the pandemic therefore contributed in this adaption, possibly also in urban regions. However, both studies showed that digital solutions do not work in all situations; this modification is not always a suitable solution in patient care. There appears to be greater potential for internal use of digital tools. They can help the FACT teams to function even if the staff members do not have face-to-face meetings on a daily basis, by saving time and increasing the use of multidisciplinary expertise. Concurrently, the FACT model states that the teams should have a base location where all staff members meet daily to ensure team feeling (17). However, our study suggests that a modification in this area could work if the teams are aware of the potential problems and have regular face-to-face meetings. Thus, the use of digital tools appears to be an innovative modification that increases the model's potential in rural areas. The teams included in the present study had established the use of digital tools in practice. Moreover, this modification appears to function well and can be repeated by other teams. Digital tools are also a crucial modification for enabling geographical spread of the staff members.

Context-adaptive planning is described as an innovative modification, because it can increase the use of multidisciplinary expertise, facilitate crisis management and intensive outreach treatment and save time. The teams have established the modification in practice, and it can be repeated by other teams. In particular, visiting several patients in one trip and planning carefully which team member goes where appear to be important elements. The Danish FACT teams also addressed the travel distance problem by visiting several patients in one journey and co-ordinating tasks internally (13). Effective use of the time spent in the car also mitigates the problem of long travel distance and could be a resource-efficient modification for rural FACT teams. However, context- adaptive planning might be more an elaboration than a modification of the model. Planning work- days are important in all FACT teams, even though rural teams seem to benefit on increased focus on this element. Despite differences between rural areas (9, 48, 75), these adaptations could be relevant to other FACT teams and similar rural teams and can be considered innovative modifications of the FACT model. The model was not developed for rural contexts as one can find in Norway, which may imply the need to create a modified rural FACT model. Moreover, modifications or elaborations, such as context-adaptive planning, digital tools, some delegations of tasks and different geographical locations of staff, might also be useful in more urban regions. Reducing FACT boards meetings and high levels of part- time positions are though adaptions that might create challenges in urban areas.

### Strengths and Limitations

To increase the relevance of the present study (76), it was designed to potentially improve practice in FACT and other multidisciplinary or outreach teams in rural areas, both in Norway and other countries. Five team leaders from different rural FACT teams were included. The participants provided rich and in-depth data on the topic (77), and were regarded as having strong information power (78), because of their close knowledge of FACT team‘s challenges and modifications in rural conditions. However, including service users or other team members in the study could have provided even richer information. And there is a need for more in- depth studies to see how the different items in the FACT fidelity scale are affected by the rural context, also for FACT teams using the FACT fidelity scale 2017 (79). This is especially important since the scale has developed in the period from 2010 to 2017, on items that could affect challenges and adaptions in rural contexts.

Despite online interviews, the data were considered to be of high quality. To increase credibility (80), the same interview guide was used in all interviews and participants were often asked to provide specific examples of the issues that they described. Some participants had worked in rural areas for many years, which might have made it difficult to explain the peculiarities of such areas. However, they participated in networks with both urban and rural FACT teams, which made it easier to discuss the particular features of a rural context. Before data collection, thoughts about expected findings were written down. Some findings were unexpected, and to strengthen the analysis, these findings were carefully followed up (69). Examples of such findings are that digital platforms were used to a very large extent and that handling of crises was described as highly challenging.

To validate and increase transferability, contextual descriptions are important (70), and characteristics of the FACT teams have therefore been emphasized. However, more specific contextualization could have reduced confidentiality. Quotations should also be contextualized (70), but to maintain confidentiality, they were not contextualized as to which team they represented. As recommended in the checklist of the Consolidated Criteria for Reporting Qualitative Research (81), we used quotations from different participants, and all of them were cited.

The described challenges and adaptions can also be relevant for other FACT teams or services working in rural areas and to some degree also in urban regions. This makes the article relevant also when implementing similar innovations in health services. This also points to the fact that the challenges and adaptions can't be stated unique for FACT teams only.

## Conclusion

Rural and remote contexts challenge the potential of the FACT model. Several key elements of the model are difficult to achieve because of low population densities and long travel distances. Remote locations make it challenging for the FACT teams to conduct intensive outreach and provide most services. They also make it more difficult to handle crises and to work in line with the multidisciplinary team approach. Some challenges appear to have been handled and reduced through modifications to the FACT model, while other challenges could not be addressed by the teams themselves. This was particularly evident when the teams had to wait for many hours for the emergency services in crisis situations. Some of the modifications also appear to make it more difficult to meet the requirements of the FACT model regarding a multidisciplinary shared caseload approach. This applies especially to part-time positions and reduction in the number of FACT board meetings. The rural context also means that the teams must have a lower caseload than outlined in the model. On the one hand, this modification increases the potential of the FACT model, but on the other hand, it makes the teams less cost-effective. This can in turn make rural FACT teams less sustainable over time.

The present study also indicates that some of the rural FACT teams used innovative modifications that could increase the model's potential in rural contexts. Intermunicipal collaboration and use of digital tools are modifications that enabled the teams to comply better with the key elements of the FACT model. Increased focus on context-adaptive planning might also be favorable, while some delegation of tasks to the municipal services and different locations of the staff members could work under certain conditions. Despite this, if distances are very long and population density very low, the central elements model seems to be difficult to accommodate. If key requirements of the model such as the multidisciplinary shared caseload approach, intensive outreach, crisis management and self-provision of most services are considerably weakened, the implementation of FACT in rural and remote areas will scarcely resemble the FACT model.

## Data Availability Statement

The datasets generated to this study are not publicly available due to concerns regarding patient/participant anonymity.

## Ethics Statement

Ethical review and approval was not required for the study on human participants in accordance with the local legislation and institutional requirements. The patients/participants provided their written informed consent to participate in this study.

## Author Contributions

KT performed the interviews, analyzed, and wrote the article. ASL, KA, MR, AL, and SO participated in the revision of the article. All authors read through and approved the final article.

## Funding

The study was funded by the Norwegian Research Council, Number 288722.

## Conflict of Interest

The authors declare that the research was conducted in the absence of any commercial or financial relationships that could be construed as a potential conflict of interest.

## Publisher's Note

All claims expressed in this article are solely those of the authors and do not necessarily represent those of their affiliated organizations, or those of the publisher, the editors and the reviewers. Any product that may be evaluated in this article, or claim that may be made by its manufacturer, is not guaranteed or endorsed by the publisher.

## Abbreviations

COVID-19: Coronavirus disease 2019
FACT: Flexible assertive community treatment
NAV: The Norwegian Labor and Welfare Organization
GP: General practitioners
ACT: Assertive community treatment.
